# Case Report: Identification Pathogenic Abnormal Splicing of *BBS1* Causing Bardet–Biedl Syndrome Type I (BBS1) due to Missense Mutation

**DOI:** 10.3389/fgene.2022.849562

**Published:** 2022-05-27

**Authors:** Kai Yan, Yixi Sun, Yanmei Yang, Bei Liu, Minyue Dong

**Affiliations:** ^1^ Department of Reproductive Genetics, Women’s Hospital, School of Medicine, Zhejiang University, Hangzhou, China; ^2^ Key Laboratory of Reproductive Genetics (Zhejiang University), Ministry of Education, Hangzhou, China

**Keywords:** Bardet–Biedl syndrome type I, BBS1, alternative splicing, whole-exome sequencing (WES), premature termination codon (PTC), nonsense-mediated decay (NMD)

## Abstract

Conventionally, protein features affected by missense mutation was attributed to destroy an important domain with amino acid alternation, and it was difficult to clearly specify the pathogenicity of a novel missense mutation. Nevertheless, the associations between missense mutations and abnormal splicing are nowadays increasingly reported. Rarely, some missense mutations, locating at the non-canonical splicing sites, are observed to damage the splicing process. In this study, a couple has three adverse pregnancy history that the affected fetus presented typical polydactyly, renal abnormalities, and cerebral ventriculomegaly. To identify its genetic etiology, whole-exome sequencing (WES) was performed and a missense mutation c.1339G > A was identified, which was located at the non-canonical splicing sites of the *BBS1* gene. Then, reverse transcription polymerase chain reaction was carried out and demonstrated extra 115bp originating from intron 13 cut into cDNA, which generated a predicted premature termination codon (PTC) in the BBS1 protein. Further expression analysis by using real-time reverse-transcribed PCR confirmed the occurrence of nonsense-mediated decay (NMD). Therefore, the pathogenicity of the missense mutation c.1339G > A was explicit and our study helped to extend the spectrum of pathogenic mutations in Bardet–Biedl syndrome type I.

## Introduction

Bardet–Biedl syndrome (BBS) is a genetically heterogeneous autosomal recessive ciliopathy initially reported by Georges Bardet and Arthur Biedl. The primary clinical features of BBS include retinal degeneration/dystrophy (RD), obesity, polydactyly, hypogonadism and genital abnormalities, intelligence disability, renal abnormalities, and behavioral dysfunction. Some secondary clinical features have also been reported (Khan et al., 2016).

The incidence of BBS varies widely across the population, ranging from nearly 1 in 125,000–160,000 in North America and Europe (Ajmal et al., 2013) and 1 in 156,000 in North Africa (M'Hamdi et al., 2011) to a high morbidity such as 1 in 17,000 among the Bedouins in Kuwait (Farag and Teebi, 1989), 1 in 13,000 in Newfoundland (Brinckman et al., 2013), and 1 in every 3,700 individuals in the Faroe Islands (Ajmal et al., 2013; Hjortshoj et al., 2009). This phenomenon is caused by the founder effect in these particular populations (Fattahi et al., 2014). In addition, the high rate of consanguinity and the geographic isolation also play a vital role (Farag and Teebi, 1989; Moore et al., 2005).

Thus far, 21 BBS genes located on different chromosome have been identified (M'Hamdi et al., 2014), the majority of which are involved in the formation and proper functioning of a multi-subunit complex (BBSome). The stable complex participates in signaling receptor trafficking to cilia, which is required for proper cilia functions (Khan et al., 2016). These identified BBS genes can explain approximately 80% of the clinical examined affected. *BBS1* is the most common gene accounting for about 23% of the cases in Europe and North America (Forsythe and Beales, 2013). The typical inheritance pattern of this syndrome is autosomal recessive. However, it has been hypothesized that a more intricate and provocative mode called “triallelic inheritance” or “digenic trait” played an important role in 10% of the BBS families (Katsanis, 2004; Forsythe and Beales, 2013).

In this study, we reported a couple who suffered from three continuous pregnancies with fetal polydactyly, renal abnormalities, and cerebral ventriculomegaly. We sequenced the whole exome of the couple, and only an unclassified missense mutation *BBS1*: c.1339G > A was found both in male and female samples. Further functional analysis clarified the pathogenicity, pathogenesis, and classification of this mutation, which expanded the mutation spectrum. Meanwhile, it enhanced our understanding that the aberrant splicing effect caused by silence or missense mutations falling in the last nucleotide of exon cannot be ignored.

## Patients and Methods

### Case Presentation

**FIGURE 1:**
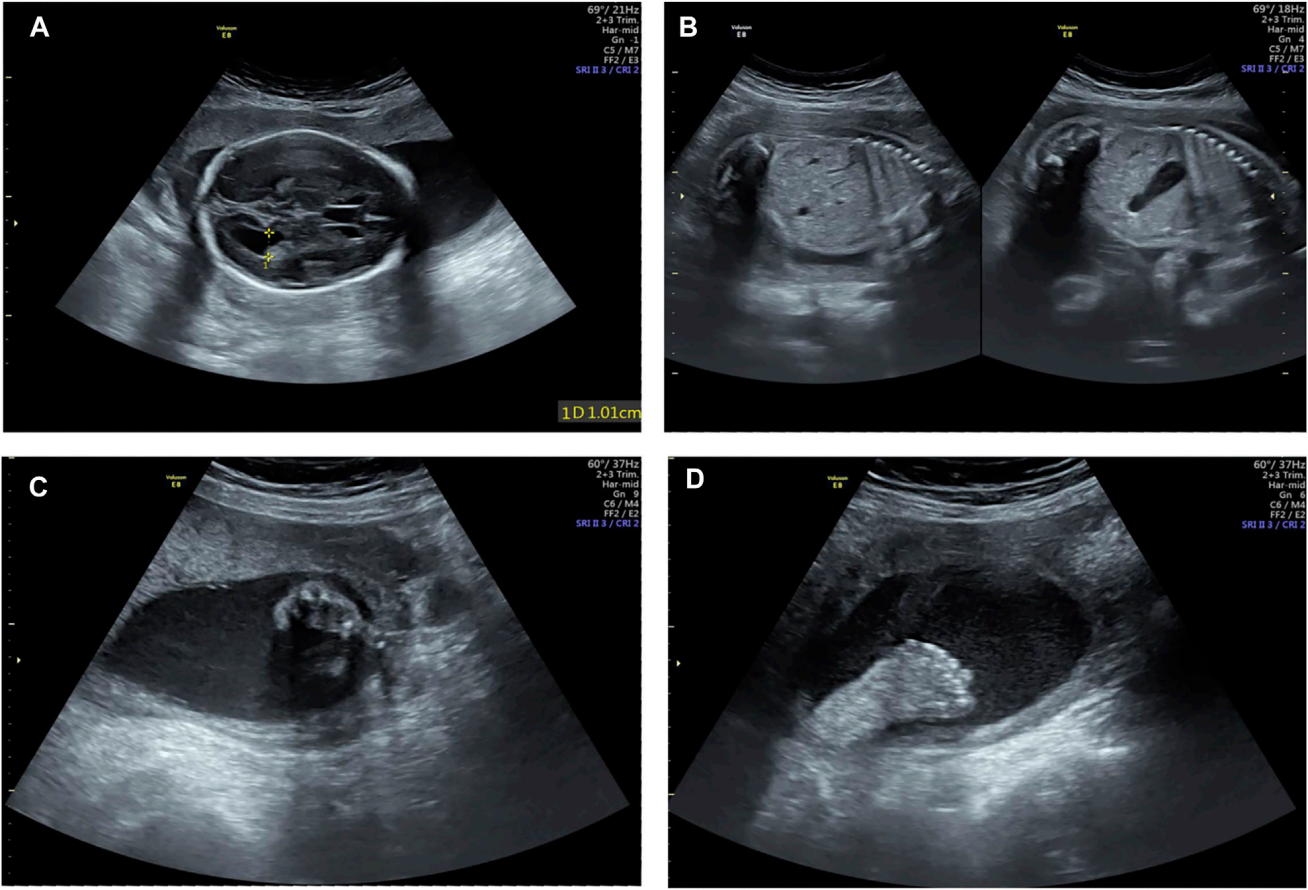
Examination image of the fetus. Fetal ultrasound scan showed fetus malformation including lateral ventricle widening **(A)**, bilateral renal echo enhancement **(B)**, and polydactyly **(C,D)**.

A healthy couple was referred to our clinic for genetic counseling after suffering from three voluntary terminations of pregnancy with congenital fetus malformation. The fetus malformation included polydactyly and renal abnormalities (Figure 1). Regrettably, the sample of fetus was not available. To explore the underlying genetic cause, conventional cytogenetic studies were performed, and single-nucleotide polymorphism (SNP)-based chromosomal microarray analysis (CMA) and whole-exome sequencing (WES) for further detection were employed subsequently.

### Karyotype and SNP Array

The karyotype analysis of peripheral blood was determined by conventional karyotyping of at least 30 blood lymphocytes, which were arrested at metaphase by colchicine. G-banding karyotypes of cultured cells was performed at the 320–400 band level with a resolution of around 20 Mb. SNP array was performed by the CytoScan™ HD array (Affymetrix, United States) according to the manufacturer’s instruction, with around 2,600,000 markers including 750,000 SNP probes and 1,900,000 non-polymorphism probes for comprehensive coverage of whole-genome. Data were analyzed by the Chromosome Analysis Suite (ChAS) software (Affymetrix, Santa Clara, CA) based on the GRCh37/hg19 assembly. The reporting threshold of the copy number result was set at 500 kb with a marker count of ≥50 for gains and at 200 kb with a marker count of ≥50 for losses.

### Genomic DNA and RNA Extractions

DNA from whole blood was extracted using the QIAamp DNA blood mini Kit (QIAGEN, 51104). Peripheral blood mononuclear cells (PMBCs) were isolated by Ficoll density gradient separation. Total RNA was extracted from PMBCs using TRIzol (Takara, Japan). Extracted total RNAs were reverse-transcribed using the RT Kit (Takara, Japan).

### Whole-Exome Sequencing

The main step of WES was provided by Veritas Genetics Institute. Genomic DNA was extracted from peripheral blood samples using a Lab-Aid 824 Nucleic Acid Extraction Kit (Zeesan Biotech) according to the manufacture procedures, 200 ng of gDNA were sheared to 150–200 bp target DNA fragments for library preparation. Libraries were constructed with pre-capture library, library hybridization, and capture with SureSelect V6 probes (Agilent), post capture library amplification, and purification using the SureSelect XT HS Target Enrichment System (Agilent). After purification, the indexed libraries were quantified and qualified with Qubit 2.0 Fluorometer (Thermo Fisher) and Qsep100 (Bioptic). All samples were diluted and pooled for multiplexed sequencing on NovaSeq 6000 (Illumina).

For prediction of the influence on the splicing, the online software SpliceAI (https://spliceailookup.broadinstitute.org/) was used.

### Reverse-Transcribed PCR and Real-Time Reverse-Transcribed PCR

Reverse-transcribed PCR was carried out to identify the splicing alternations. Then, the PCR products were analyzed by 1.5% agarose gel electrophoresis and sequenced as previously described. Real-time reverse-transcribed PCR was performed using TB Green™ Premix Ex Taq™ II (Tli RNaseH Plus) (Takara, RR820A) with the manufacturer’s instructions. Relative mRNA expression was normalized to β-ACTIN and analyzed by the ΔΔCt method.

## Results

### Identification of Heterozygous *BBS1* Missense Variant c.1339G > A

**FIGURE 2:**
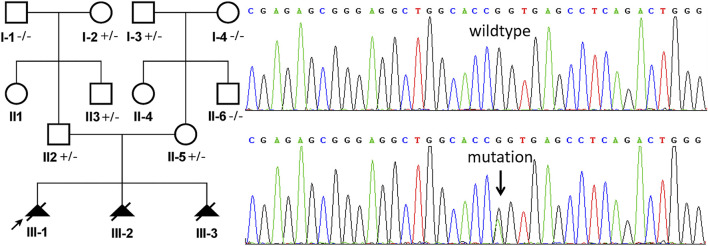
Sanger sequencing analysis of genomic DNA from family members. The genotypes of *BBS1* were c.1339G > A heterozygous in II:2 (husband, Maternal Origin) and II:5 (wife, Paternal Origin). The mutation is indicated by the arrows.

A heterozygous missense mutation (chr11: 66294278, NM_024649.5, c.1339G > A) was identified in the exon 13 of the *BBS1* gene both in the male and female samples by WES, which was confirmed by Sanger sequencing (Figure 2). This mutation has been reported previously and was classified as conflicting interpretations of pathogenicity. According to the genome aggregation database (gnomAD) v2.1.1, the allele frequency (AF) in East Asian, African, and European (non-Finnish) was 0.0002506, 0.0001385, and 0.00008011, respectively (the AF matched with the evidence of PM2). Moreover, the software prediction was inconsistent (PolyPhen2, benign; SIFT, tolerated; Mutation Taster, disease causing). According to the guidelines of the American College of Medical Genetics and Genomics (ACMG), available evidence are not sufficient to support this mutation as “pathogenic” or “likely pathogenic,” but there are no other causative mutations.

### Confirmation of the Aberrant Splicing

**FIGURE 3:**
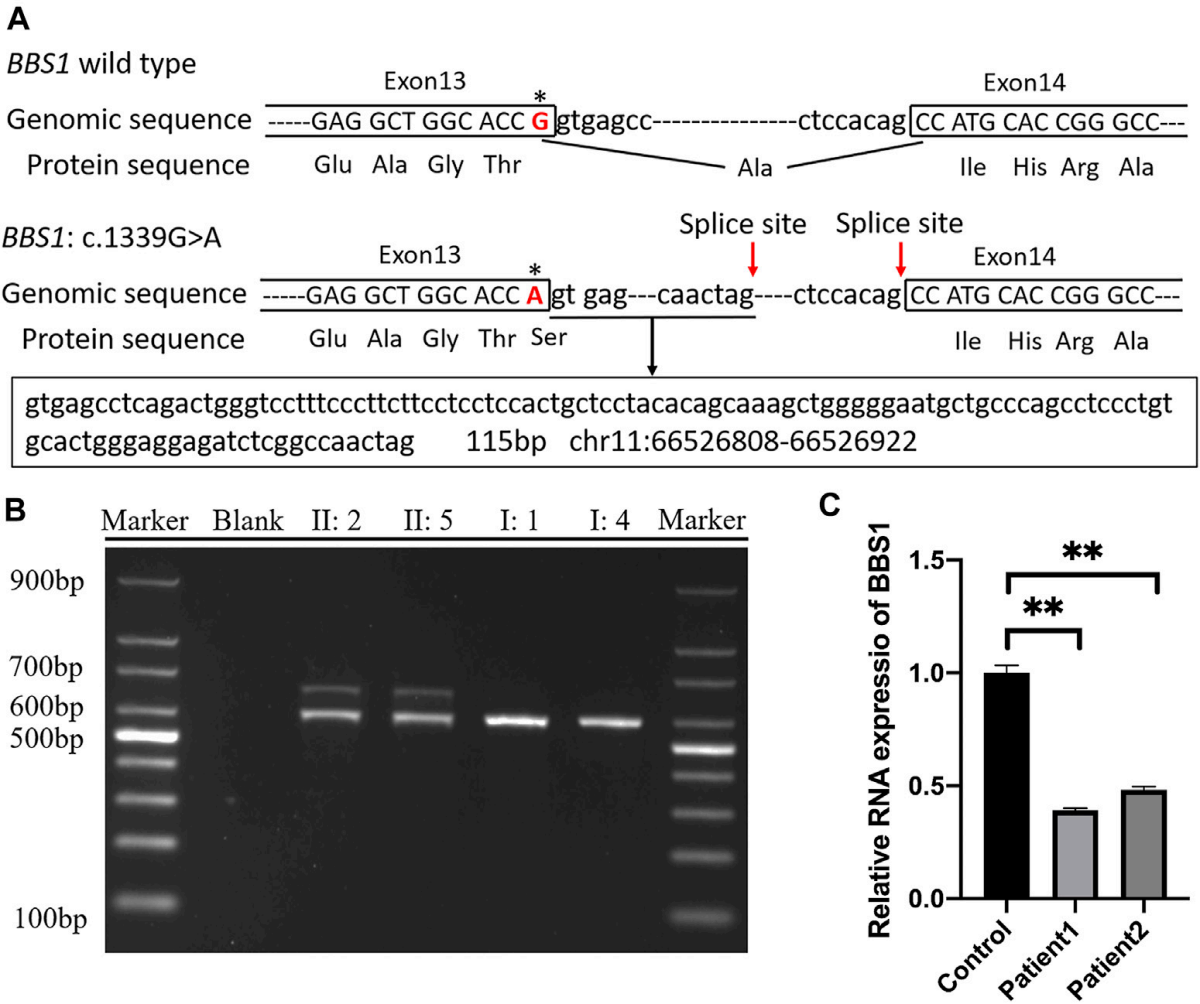
**(A)** Schematic representation of exon 13, intron 13, and exon 14 organization in *BBS1*; **(B)** RT-PCR analysis of exons 13–15 of the *BBS1* cDNA from peripheral blood mononuclear cells (PMBCs). Agarose gel electrophoresis of RT-PCR products generated from heterozygous II:2, II:5, I:1, and I:4. **(C)** Real-time RT-PCR of carrier couple and the control lymphoblast cells, revealing half of the *BBS1* transcript in the carriers’ PBMCs. Error bars indicate SE of the means; *p*-value< 0.05 (unpaired *t* test).

The online software SpliceAI suggested the score of donor loss was 0.83. Therefore, transcript analysis was performed for confirmation of aberrant splicing. RT-PCR was performed using primers designed to amplify exons 13–15 in BBS1 mRNA. The agarose gel electrophoresis of PCR products results showed that both couples had long/short bands with approximately 100 base pair discrepancies, while sole band corresponding to the normal control (Figure 3B). TA-cloning sequencing of the amplified fragment was carried out and BLAST search results in NCBI showed that the mutation led to extra 115 bp originating from intron 13 into cDNA so that the terminal codon generated in advance (Figure 3A).

### The Missense Mutation in the *BBS1* Gene Induces Nonsense-Mediated Decay (NMD)

It was explicit that the premature termination codon (PTC) was always considered harmful for protein function. The rule for termination codon position has broad implications given that, in principle, any intron located more than 50–55 nucleotides downstream of a termination codon could mediate a reduction in mRNA abundance. To validate the deduction, real-time PCR was performed and revealed half of the *BBS1* transcript in the carriers’ PBMCs when compared with the control (Figure 3C), which confirmed the occurrence of NMD.

## Discussion

Comparing with prokaryotes, eukaryotes exhibit a much higher degree of variety and complexity, which greatly attributes to the function of alternative splicing. Up until now, 127 splice variants of *BBS1* have been included in the human genome mutation database (HGMD^®^ Professional 2021.2), most of which located in the intron region near the exon boundary, except for two silence mutation c.1110G > A, p. (Pro370 = ), c.1695G > A, p. (Lys565 = ) and one missense mutation c.479G > A, p. (Arg160Gln). Missense mutations in important domains alter amino acid sequences, which may contribute to the changes of protein features. It is challenging to define the pathogenicity of this novel missense mutation. Therefore, we still do not fully understand some of the missense mutations detected by WES. In this study, we employed WES to explore the genetic cause of a suspicious BBS carrier couple and a missense variant *BBS1*: c.1339G > A was detected, which was first reported by Taylor's study (Taylor et al., 2017) in 85 children with inherited retinal disease. Although the identified missense mutation has been included in the ClinVar database and human genome mutation database (HGMD^®^ Professional), its classification was still disputable because available evidence (PM2 and PP4) only support the classification of VUS. To the author’s knowledge, it was reported in Chinese population for the first time and was the original study about its function. With transcript analysis, we proved this missense mutation created a new 5′ splice site that led to extra 115 bp originating from intron 13 into cDNA, which generated a predicted premature termination codon (PTC) in BBS1 protein (NP_078925). According to the recommendations for interpreting the loss of function PVS1 ACMG/AMP variant criterion (Abou et al., 2018), the aforementioned results of the *in vitro* experiment provide the PVS1 evidence for this mutation. However, there were still some limitations of this study. RNA has tissue specific expression patterns. We attempt to see whether other tissue or cell type from the mutation carrier has the same consequence. Oral mucosa cells from saliva were considered as the applicable materials. Bernoulli experiment for three times failed, and cells from other tissue were difficult to acquire unless invasive operation.

Furthermore, the symptoms of BBS were variable, which include six primary features [retinal degeneration, truncal (central) obesity, postaxial polydactyly, hypogenetelism, intelligence disability, and renal abnormalities] and some secondary features (include 12 kinds of features). These symptoms are not always present in all BBS patients. The disorder of cilia as BBS had a significant overlap of clinical features and genetic overlap within the BBS and other ciliopathies such as Alström and McKusick–Kauffman syndromes or Meckel Gruber syndromes. The clinical diagnosis of BBS requires that a patient displays either four primary features or a combination of three primaries and two secondary features, which can effectively differentiate this syndrome from other phenotype overlapped syndromes (Beales et al., 1999). As the previous study showed (Fu et al., 2018; Lord et al., 2019; Petrovski et al., 2019), the truth that for the same illness, the molecular diagnostic rate for likely pathogenic variants associated with malformed fetal phenotypes is slightly lower than the rates reported in larger scale clinical exome sequencing, studies on postnatal patients might be due to the fact that prenatal phenotypic evaluation is limited and genotype–phenotype assessments are extremely difficult. In this case, the ultrasound examination suggested polydactyl, lateral ventricle widening, and bilateral renal echo enhancement that were consistent with BBS. However, it still did not meet the diagnostic criteria because most symptoms listed before can only be detected postnatally even until childhood. Even so, the available evidence still supported the clinical diagnosis of BBS and matched with the evidence of PP4. Therefore, according to the ACMG/Association for Molecular Pathology (AMP) guideline (Richards et al., 2015), this mutation can be classified as “Pathogenic” (PVS∗1 + PM∗1 + PP∗1).

As is well-known, the incidence of BBS varies widely worldwide. The global average AF value of c.1339G > A was 0.00004609, while it was 0.0002506 in East Asia, which was significantly higher than other ethnic groups. In addition, the non-consanguinity couple in this pedigree carried the same mutation in BBS1. Ethnic specific variants had been reported for other autosomal recessive disease previously (Alanay et al., 2010; Cabral et al., 2012; Essawi et al., 2018). Therefore, we cannot rule out a founder effect of this mutation in Chinese population or a relatively isolated region subjectively. Furthermore, the AF and incidence of this *BBS1* variant can be estimated within large scale exon data and confirmed whether it was a founder mutation or do haplotype analysis and mutation dating as follow-through (Zhu et al., 2021). The identification of ethnic specific founder mutation can help to determine screening strategy for disease prevention and facilitate genetic counseling. The *BBS1*: c.1339G > A was a defined pathogenic variation that should be included in expended carrier screening so that this severe condition can be prevented at an early stage.

Our study provides further evidence that WES facilitates genetic diagnosis of fetal structure abnormalities, allowing for more accurate detection of etiology. Performing WES on trios was benefit for detecting *de novo* or compound heterozygous variants, and reducing the percentage of VUS. In turn, *de novo* variants can be used to identify potential novel disease-causing genes. In this case, the samples of abnormal fetus were unavailable so that we had to obtain suspected variants from the couple. We found the causative mutation fortunately and identified the correlation between genotype and phenotype. However, the application of this indirect analysis was limited. Therefore, the proband’s sample is indispensable as the direct evidence for genetic etiology identification, which needs to be better implemented, especially in primary care institutions.

## Conclusion

To our knowledge, it is the first study to determine the pathogenicity and pathogenesis of the missense mutation *BBS1*: c.1339G > A. Our work helped to expand the spectrum of pathogenic mutations in the *BBS1* gene and the expanded carrier screening. At the same time, it is an alerting sign that the aberrant splicing effect caused by silence or missense mutations falling in the last nucleotide of exon cannot be ignored.

## Data Availability

The datasets for this article are not publicly available due to concerns regarding participant/patient anonymity. Requests to access the datasets should be directed to the corresponding author.
